# Fatal Hæmorrhage from a Free Incision of the Gums

**Published:** 1846-12

**Authors:** 


### ARTICLE VI.

### *Fatal Hæmorrhage from a Free Incision of the Gums.*

Mr. Anderson relates the following case, in the *London Lancet:* I was called to visit a child six months old; it had been delicate from the first month of its life, but afterwards became stout and healthy, until the day before I saw it. I found it laboring under symptoms of pain in the abdomen, as frequent fits of crying, attended with a bending of the knees upon the abdominal parieties. There had been no sickness nor vomiting; the bowels were not constipated, nor was there diarrhœa; the evacuation of urine was but little diminished in quantity; there were no cerebral symptoms present; no disturbance of the respiration in the intervals free from crying; and no fever. I examined the gums, and found those over the upper central incisors a little red and tumid. I lanced these upper gums somewhat freely; they bled, the child took the breast, and every trace of blood soon disappeared. At that time, I distinctly told the mother that the inferior gums did not require to be lanced. I sent it a grain of calomel, to be taken night and morning, and a carminative aperient mixture, to be taken every three or four hours. I believe I also directed moderate fomentation of the abdomen with hot water and flannel. My prognosis, delivered to the mother, was, "I see no danger in the case, give it the medicine, and I believe it will get well, but if it *should* become worse, send and tell us."

On Saturday morning, the 30th, I received a message that the child was considered to be worse. I went over, and found no remains of abdominal symptoms, and the reason that the child was considered worse was, that it had become unusually sleepy. I had not administered any sedative, and was uncertain whether this somnolence was the result of the relief of the abdominal pain, or the first manifestation of cerebral symptoms. Time would solve the former supposition, and fearing the latter, I directed that the head should be constantly covered with a cloth wrung out of cold water, and the medicine continued.

On Monday, June 1st, I went over before breakfast, and found the child awake and lively, and had no hesitation in saying that it would soon be well.

Thursday, June 4th, a messenger was sent to say that the the parents were uneasy about the child's body, (the abdomen,) which had swelled, and they requested that it should be seen. I was from home, and Mr. Jones promised to go directly; he did not, however, see the child until after eleven at night, although he received the message before ten in the morning.

I have been told that he paid little or no attention to the abdomen, but urged the necessity of lancing the lower gums, notwithstanding the mother's objection, and that she urged that I had so plainly told her they did not require it. He lanced them, and they bled profusely; he saw the hæmorrhage, yet he went away, only remarking, "it will do the child good, and I will send it some stronger medicine."

Friday, June 5th.—I went to the child in consequence of receiving a note, which stated, that it had been bleeding all night and was dying. Upon entering the room, I perceived the contents of the stomach, which had just been ejected on to the floor, and in which was a coagulum of not less than two ounces of blood. I was told, that once previous to that, a similar quantity had been thrown up, and that much blood had passed in five or six evacuations from the bowels since the gums had been lanced the night before. The urine had been voided as usual; the abdomen was slightly tympanitic, and an umbilical hernia had protruded a little. This was the condition of the abdomen which had occasioned the uneasiness of the parents. The pulse was perceptible at the wrist, and the extremities were warm as usual, but the entire surface of the body was much blanched. The respiration was so hurried that it was the first thing I observed; there was also a disposition to sleep; it could not be said that the child was faint. Upon examining the gum I found an incision extending about an inch or an inch and a quarter in length on the upper border of the lower jaw. It appeared to have been begun at about the site of the outer edge of the right inferior lateral incisor tooth; to have passed with the curve of the jaw *on* the alveolus for about a quarter of an inch, then to have descended on the posterior aspect of the site of the two central incisors, separating the gum from the alveolus and to have been brought over the site of the outer edge of the left lateral incisor, transversely across the alveolus. It was bleeding freely from its entire extent, the blood, issuing out of the incision into the mouth, was swallowed, and it had not the appearance of arterial blood. I endeavored to suppress the hæmorrhage by the application of styptics, as alum in powder and in solution; the nitrate of silver; the application of cold to the chin, &c., but principally by a firm compression of the gum against the bone, continued with little intermission for two hours, but with no effect. The impatience of restraint, the crying and struggling manifested by infants in general, were in this case much opposed to the success of the means employed. I had at one time, however, nearly succeeded, as it bled only from the two extreme points, but a fit of crying brought back the hæmorrhage with renewed vigor.

Having passed several hours with the child unavailingly, I left it at near midnight and returned home, leaving word at Mr. Jones' house that *he* should go to see it. He was not at home, but went about three in the morning of Saturday. I did not see the child afterwards, but I heard on the Monday that it had died on the Sunday morning.

These, sir, are the details of this case, related to the utmost of my ability with an undeviating and an *unflinching* adherence to the truth, as free from exaggeration as from extenuation. I solicit an insertion of it in the pages of the LANCET as early as conveniently possible, and I abstain from comment because I believe it may be undertaken by persons who consider themselves more competent. No one, however, but myself could furnish a report of the case.

